# Genetic variants for prediction of gestational diabetes mellitus and modulation of susceptibility by a nutritional intervention based on a Mediterranean diet

**DOI:** 10.3389/fendo.2022.1036088

**Published:** 2022-10-13

**Authors:** Ana Ramos-Levi, Ana Barabash, Johanna Valerio, Nuria García de la Torre, Leire Mendizabal, Mirella Zulueta, Maria Paz de Miguel, Angel Diaz, Alejandra Duran, Cristina Familiar, Inés Jimenez, Laura del Valle, Veronica Melero, Inmaculada Moraga, Miguel A. Herraiz, María José Torrejon, Maddi Arregi, Laureano Simón, Miguel A. Rubio, Alfonso L. Calle-Pascual

**Affiliations:** ^1^ Endocrinology and Nutrition Department, Hospital Universitario de la Princesa, Instituto de Investigación Princesa, Universidad Autónoma de Madrid, Madrid, Spain; ^2^ Endocrinology and Nutrition Department, Hospital Clínico Universitario San Carlos and Instituto de Investigación Sanitaria del Hospital Clínico San Carlos (IdISSC), Madrid, Spain; ^3^ Facultad de Medicina. Medicina II Department, Universidad Complutense de Madrid, Madrid, Spain; ^4^ Centro de Investigación Biomédica en Red de Diabetes y Enfermedades Metabólicas Asociadas (CIBERDEM), Madrid, Spain; ^5^ Patia Europe, Clinical Laboratory, San Sebastián, Spain; ^6^ Gynecology and Obstetrics Department, Hospital Clínico Universitario San Carlos and Instituto de Investigación Sanitaria del Hospital Clínico San Carlos (IdISSC), Madrid, Spain; ^7^ Clinical Laboratory Department Hospital Clínico Universitario San Carlos and Instituto de Investigación Sanitaria del Hospital Clínico San Carlos (IdISSC), Madrid, Spain

**Keywords:** genetic risk variants, genetic polymorphisms, gestational diabetes mellitus, single nucleotide polymorphisms, SNPs, Mediterranean diet, nutritional intervention

## Abstract

**Hypothesis:**

Gestational diabetes mellitus (GDM) entails a complex underlying pathogenesis, with a specific genetic background and the effect of environmental factors. This study examines the link between a set of single nucleotide polymorphisms (SNPs) associated with diabetes and the development of GDM in pregnant women with different ethnicities, and evaluates its potential modulation with a clinical intervention based on a Mediterranean diet.

**Methods:**

2418 women from our hospital-based cohort of pregnant women screened for GDM from January 2015 to November 2017 (the San Carlos Cohort, randomized controlled trial for the prevention of GDM ISRCTN84389045 and real-world study ISRCTN13389832) were assessed for evaluation. Diagnosis of GDM was made according to the International Association of Diabetes and Pregnancy Study Groups (IADPSG) criteria. Genotyping was performed by IPLEX MassARRAY PCR using the Agena platform (Agena Bioscience, SanDiego, CA). 110 SNPs were selected for analysis based on selected literature references. Statistical analyses regarding patients’ characteristics were performed in SPSS (Chicago, IL, USA) version 24.0. Genetic association tests were performed using PLINK v.1.9 and 2.0 software. Bioinformatics analysis, with mapping of SNPs was performed using STRING, version 11.5.

**Results:**

Quality controls retrieved a total 98 SNPs and 1573 samples, 272 (17.3%) with GDM and 1301 (82.7%) without GDM. 1104 (70.2%) were Caucasian (CAU) and 469 (29.8%) Hispanic (HIS). 415 (26.4%) were from the control group (CG), 418 (26.6%) from the nutritional intervention group (IG) and 740 (47.0%) from the real-world group (RW). 40 SNPs (40.8%) presented some kind of significant association with GDM in at least one of the genetic tests considered. The nutritional intervention presented a significant association with GDM, regardless of the variant considered. In CAU, variants rs4402960, rs7651090, IGF2BP2; rs1387153, rs10830963, MTNR1B; rs17676067, GLP2R; rs1371614, DPYSL5; rs5215, KCNJ1; and rs2293941, PDX1 were significantly associated with an increased risk of GDM, whilst rs780094, GCKR; rs7607980, COBLL1; rs3746750, SLC17A9; rs6048205, FOXA2; rs7041847, rs7034200, rs10814916, GLIS3; rs3783347, WARS; and rs1805087, MTR, were significantly associated with a decreased risk of GDM, In HIS, variants significantly associated with increased risk of GDM were rs9368222, CDKAL1; rs2302593, GIPR; rs10885122, ADRA2A; rs1387153, MTNR1B; rs737288, BACE2; rs1371614, DPYSL5; and rs2293941, PDX1, whilst rs340874, PROX1; rs2943634, IRS1; rs7041847, GLIS3; rs780094, GCKR; rs563694, G6PC2; and rs11605924, CRY2 were significantly associated with decreased risk for GDM.

**Conclusions:**

We identify a core set of SNPs in their association with diabetes and GDM in a large cohort of patients from two main ethnicities from a single center. Identification of these genetic variants, even in the setting of a nutritional intervention, deems useful to design preventive and therapeutic strategies.

## Introduction

Gestational diabetes mellitus (GDM), defined as diabetes newly diagnosed in the second or third trimester of pregnancy, and was not clearly overt diabetes prior to gestation (1), is a frequent gestational metabolic complication that has become a major public health issue. Its prevalence has significantly increased in parallel with increasing rates of obesity, older age at pregnancy, and the implementation of the International Association of the Diabetes and Pregnancy Study Groups criteria (IADPSG criteria) (2). GDM is associated with adverse maternal and neonatal outcomes and an increased risk for the future development of type 2 diabetes both in the mother and the offspring (1, 2), so strategies for early detection and prevention, and interventions to control maternal glucose levels have become a priority.

The complex underlying pathogenesis of GDM includes a specific genetic background and the effect of environmental factors. Although there is still much to be known regarding the underlying mechanisms responsible for the development of GDM, several modifiable and non-modifiable factors have been acknowledged; for instance, increased adiposity, lifestyle, ethnicity, increased maternal age, polycystic ovary syndrome or a family history for type 2 diabetes. Regarding the genetic background, several genetic polymorphisms have been identified as potentially associated with an increased risk of developing GDM, most of them overlapping with those associated with the risk of type 2 diabetes. However, there is still controversy on the true impact of genetic polymorphisms on the risk of these metabolic alterations, and whether this increased risk could be modulated by clinical interventions such as diet. In previous studies (3, 4) we found that an early nutritional intervention with a supplemented Mediterranean diet (MedDiet) reduces the incidence of GDM and, consequently, our hospital recommended the adoption of this nutritional intervention to all pregnant women.

The objective of this study is to examine the link between a set of single nucleotide polymorphisms (SNPs) associated with diabetes and GDM, according to different bibliographical references, and the development of GDM in pregnant women of different ethnicities, in the setting of a clinical intervention based on the MedDiet.

## Methods

### Study population

The study population originates from our hospital-based cohort of pregnant women screened for GDM from January 2015 to November 2017 (the San Carlos Cohort, randomized controlled trial (RCT) for the prevention of GDM registered December 4, 2013 at ISRCTN84389045 (DOI 10.1186/ISRCTN84389045) and real-world study, registered October 11^th^, 2016 at ISRCTN13389832 (DOI 10.1186/ISRCTN13389832) (3, 4) with approval by the Clinical Trials Committee of the Hospital Clínico San Carlos (July 17, 2013, CI 13/296-E and October 1^st^, 2016, CI16/442-E, respectively), and compliance with the Declaration of Helsinki). The central location of our hospital and its relatively large reference healthcare population of around 445,000 implied that our study sample could adequately represent the population living in our country.

**Figure 1:**
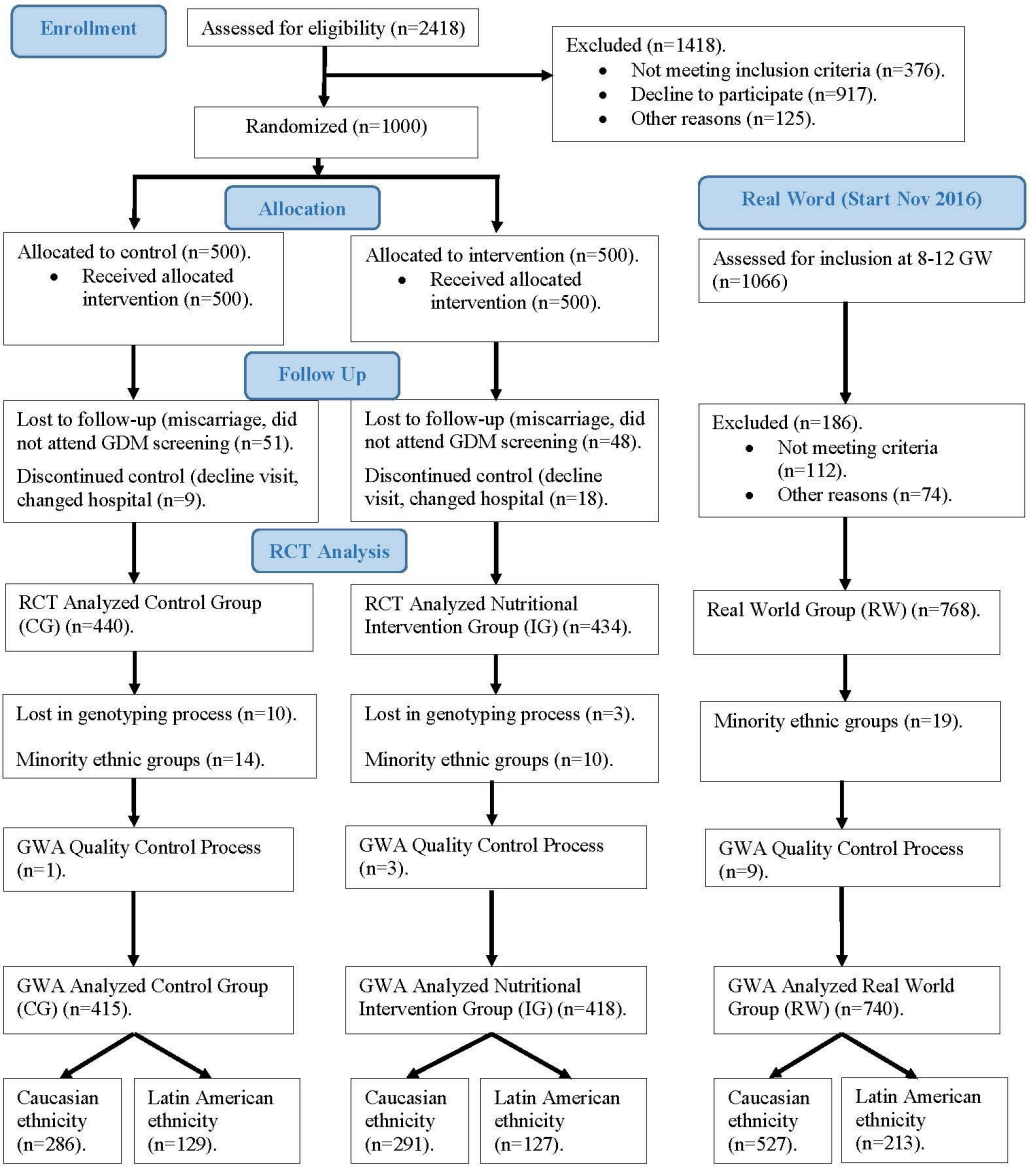
Flow diagram of women included in our study.

**Figure 1** shows the CONSORT 2010 flowchart of our study population. From January 2015 to November 2017, a total of 2418 women who attended their first gestational visit (at 8 ± 2 gestational weeks (GW), in which the first ultrasound is performed and analytical screening for chromosomal alterations is carried out), with fasting plasma glucose (FPG) < 92 mg/dL, were assessed for the clinical trial. Inclusion criteria were ≥18 years old, singleton gestation, and willingness to participate in the study. Exclusion criteria comprised gestational age at entry >14 weeks, pre-gestational diabetes, diseases affecting carbohydrate metabolism, intolerance to nuts or extra-virgin olive oil (EVOO), and medical conditions or pharmacological therapy that could compromise the effect of the intervention and/or the follow-up program. All patients included signed a written informed consent.

A sample of 1000 women was selected and randomly divided into two groups of the same size, control group (CG) and intervention group (IG), according to two nutritional intervention models. The same basic MedDiet and daily exercise habits were recommended for both groups. Participants allocated to IG received lifestyle guidance from dieticians one week after inclusion in a unique 1-hour group session. The key IG recommendation was a daily consumption of at least 40 mL of EVOO and a handful (25-30g) of pistachios. To ensure the consumption of the minimum amount recommended, women were provided with 20 L of EVOO and 4 Kg of roasted pistachios. Women in the CG were advised by midwives to restrict consumption of dietary fat, including EVOO and nuts. These recommendations are provided in local antenatal clinics as part of the available guidelines in pregnancy standard care (5). The first women was included on January 2^nd^, 2015 and the last one was included on December 27^th^, 2015. The follow up until delivery on July 2016. The study was completed by 874 women (440/434, CG/IG). This group is the initial sub-cohort of this paper.

The aforementioned RCT concluded that an early nutritional intervention with a supplemented MedDiet reduces the incidence of GDM (3). Based on these results, our hospital recommended the adoption of this nutritional intervention (i.e., MedDiet enriched with EVOO and nuts), without providing these specific products, to all pregnant women, from the beginning of gestation, in real word (4). Thus, from November 2016 onwards, every pregnant woman who attended the first gestational visit were invited to participate in our study based on the implementation of the RCT results in clinical practice. The last women included on November 30, 2017 was follow up until delivery on July 2018. In accordance with the inclusion and exclusion criteria indicated above, a new sub-cohort (real-world group, RW) was defined, with 768 samples that are included in this study.

Ethnicity of participants includes mainly Caucasian and Hispanic, as well as some minority ethnicities (Chinese, African and others). Given the characteristics of this study, samples corresponding to these minority ethnic groups were excluded. Therefore, samples from 1586 pregnant women were available and were used for this study. The characteristics of patients included in the study are displayed in **Table 1**.

**Table 1:** Main characteristics of patients included in the study.

		Gestational diabetes mellitus	
		NO	YES
		N (%)	N (%)
Ethnicity	Caucasian	915 (70.3)	189 (69.5)
	Hispanic	386 (29.7)	83 (30.5)
	Total	1301 (100)	272 (100)
Intervention nutritional group	Control (CG)	319 (24.5)	96 (35.3)
	Intervention (IG)	349 (26.8)	69 (25.4)
	Real Word (RW)	633 (48.7)	107 (39.3)
	Total	1301 (100)	272 (100)
Age (years)		33 ± 5	34 ± 5
Prior body weight (kg)		59.4 ± 9.72	62.82 ± 10.99
Prior BMI		22.47± 3.43	23.99 ± 4.01
Parity	1	567 (43.6)	117 (43.0)
	2	394 (30.3)	86 (31.6)
	3	203 (15.6)	41 (15.1)
	≥ 4	129 (9.9)	28 (10.3)
	NA	8 (0.6)	0 (0)
	Total	1301 (100)	272 (100)
Obstetric history	None	804 (61.8)	162 (59.6)
	Abortion	422 (32.4)	85 (31.2)
	GDM	28 (2.2)	10 (3.7)
	HT	14 (1.1)	1 (0.4)
	Other	33 (2.5)	14 (5.1)
	Total	1301 (100)	272 (100)

Data are presented as number and percentage for categorical values and mean ± standard deviation for quantitative values

### Patient data collection

Data regarding clinical, demographic and anthropometric characteristics was collected from medical records and follow-up visits. Specifically, we collected information on maternal age, ethnicity, gestational week at the time of the oral glucose tolerance test (OGTT), body mass index, family history of type 2 diabetes, past medical history of GDM, past obstetric history and parity, gestational weight gain, associated comorbidities, and the newborn’s birthweight.

### Diagnosis of gestational diabetes mellitus

A 2-hour OGTT with 75-g glucose was performed at 24-28 weeks of gestation. FPG levels were determined by the glucose oxidase method in fresh plasma samples. The International Association of Diabetes and Pregnancy Study Groups (IADPSG) criteria were used for the diagnosis of GDM (2).

### Genotype analysis

Genomic DNA was extracted from EDTA-stabilized blood samples taken during the OGTT using the Maxwell RSC instrument (Promega, Dubendorf, Switzerland).

Genotyping was performed by IPLEX MassARRAY PCR using the Agena platform (Agena Bioscience, SanDiego, CA). IPLEX MassARRAY PCR and extension primers were designed from sequences containing each target SNP and 150 upstream and downstream bases with AssayDesign Suite (http://agenabio.com/assay-design-suite-20-software) using the default settings. Single base extension reactions were performed on the PCR reactions with the iPLEX Gold Kit (AgenaBioscience) and 0.8µl of the custom UEP pool. The kit contains mass modified terminator nucleotides that increase the mass difference between extended UEPs, allowing for greater accuracy in genotyping. The mass difference with unmodified terminator nucleotides ranges from 9 to 40 kDa, depending on the two nucleotides compared. With the mass-modified terminator nucleotides the mass difference increases to 16–80 kDa. The single base extension reactions were cycled with a nested PCR protocol that used five cycles of annealing and extension nested with a denaturation step in a cycle that was repeated 40 times for a total of 200 annealing and extension steps. The goal was to extend nearly all of the UEPs. Following single base extension, the reactions were diluted with 16µl of water and deionized with 6 ng of resin. After deionizing for 20 min the reactions were dispensed onto SpectroChipArrays with a Nanodispenser (Agena Bioscience). The speed of dispensation was optimized to deliver an average of 20 nl of each reaction to a matrix pad on the SpectroChip. An Agena Bioscience Compact MassArray Spectrometer was used to perform MALDI-TOF mass spectrometry according to the iPLEX Gold Application Guide. The Typer 4 software package (Agena Bioscience) was used to analyze the resulting spectra and the composition of the target bases was determined from the mass of each extended oligo. These panels were designed in collaboration with PATIA and Genotyping was performed at the Agena platform located at the Epigenetics and Genotyping laboratory, Central Unit for Research in Medicine (UCIM), Faculty of Medicine, University of Valencia, Valencia, Spain.

### Selection of SNPs

The 110 single-nucleotide polymorphisms were based on literature references (6–12). Specifically, SNPs were prioritized according to the results of large meta-analysis of genome-wide association studies (GWAS) performed in European and other populations, and with the presumption that their effects can be extrapolated and generalized, and that large sample sizes allow solid estimations of the true size effect. Allele frequencies were considered to maximize the SNPs’ predictive power (effect size x allele frequency). In addition, significant SNPs identified in smaller association studies were also included. As a result, the selected SNPs for analysis fulfilled the following criteria: odds ratio (OR) >1.2, Rare Allele Frequency (RAF) >0.20 and Association Statistical Significance of *p <*1 × 10-5 (**Supplementary Table 1**).

### GWA quality control

Quality control steps removed participants with a high missing genotype rate (MIND >5%, 13 samples), removed SNPs with a high missing genotype data (GENO > 5%, 1 variant), removed SNPs due to Hardy-Weinberg exact test (HWE, p < 1 × 10^−6^, 7 variants), and removed SNPs due to allele low frequency threshold (MAF < 5%, 4 variants). As a result, our data warehouse included 1573 women and 98 SNPs, with a total genotyping rate of 0.996544 (**Supplementary Table 1**).

### Statistical analysis

Statistical analyses regarding patients’ characteristics were performed in SPSS (Chicago, IL, USA) version 24.0. Data are presented as mean ± standard deviation or median and interquartile range according to the normality of their distribution. χ2 test was used to compare qualitative characteristics and quantitative characteristics were assessed with Student’s t-test. A two-sided *p*-value <0.05 was considered statistically significant.

The association between each SNP and GDM risk was evaluated by genetic binary logistic regression models. All genetic association tests were performed using PLINK v.1.9 and 2.0 software (13). Specifically, we used the following models and tests: ADDITIVE model – test ADD; DOMINANT model – test DOM; RECESSIVE model – test REC and HETHOM model -test HOM and HET.

In all the logistic regression models, a variable was added to represent the nutritional intervention group [GROUP]. We defined this variable with values 1, 2 and 3 corresponding respectively to the CG, IF and RW groups of **Figure 1**. The reference group for the logistic regression model was the CG group.

The analysis was carried out by stratifying the sample by ethnicity, according to the two categories present in the data: Caucasian (CAU) and Hispanic (HIS). The allele indicated in the previous literature was taken as the reference allele (REF). In the logistic regression models, the minor allele (A1) was always taken as the base category, meaning that it can be a risk allele when OR > 1 or a protective allele when OR < 1. For each test of a model, the corresponding *p*-value was obtained using the PLINK software. As false discovery rate control (FDR), we started with the set of *p*-values and then we calculated the *q*-values (i.e. minimum FDR incurred when calling a test significant) and *lfdr*-values (*local false discovery rate*, i.e. the empirical Bayesian posterior probability that the null hypothesis is true, conditional on the observed *p-*value) using the *qvalue* package (version 2.24.0) of R software (version 4.1.2) (14), with smoother method option and adjustment of lambda parameter in the interval 0.01-0.95 with increment of 0.01 (14). As association significance criteria we used the following thresholds: *p-*value ≤ 0.05, *q*-value ≤ 0.05, *lfdr*-value ≤ 0.1.

#### Bioinformatics analysis

We mapped each SNP to its nearest corresponding protein-coding gene and then we performed gene ontology (GO) enrichment analysis and protein–protein interaction (PPI) analysis for the set of SNPs that reached significance in any of the criteria indicated above. The analysis was performed using STRING, version 11.5 (15).

## Results

### Patient data and SNP data

Quality controls retrieved a total 98 SNPs and 1573 samples, 272 (17.3%) with GDM and 1301 (82.7%) without GDM. 1104 (70.2%) were Caucasian (CAU) and 469 (29.8%) Hispanic (HIS). 415 (26.4%) were from the control group (CG), 418 (26.6%) from the nutritional intervention group (IG) and 740 (47.0%) from the real-world group (RW). Women’s main demographic and anthropometric characteristics are represented in **Table 1**. **Table 2a** CAU and **2b** HIS show the main characteristics of the variants for the Caucasian and Hispanic ethnicities, respectively.

**Table 2:** CAU Characteristics of variants. CAUCASIAN.

CHROM	LOCUS	POS	ID	REF	ALT	A1	A1_CT	ALLELE_CT	A1_CASE_CT	A1_CTRL_CT	CASE_ALLELE_CT	CTRL_ALLELE_CT	CASE_NON_A1_CT	CASE_HET_A1_CT	CASE_HOM_A1_CT	CTRL_NON_A1_CT	CTRL_HET_A1_CT	CTRL_HOM_A1_CT	A1_FREQ	A1_CASE_FREQ	A1_CTRL_FREQ	OBS_CT
1	MTHFR	11794419	rs1801131	T	G	G	630	2172	103	527	374	1798	95	81	11	437	397	65	0.290	0.275	0.293	1086
1	MTHFR	11796321	rs1801133	G	A	A	847	2204	157	690	376	1828	63	93	32	339	460	115	0.384	0.418	0.377	1102
1	PROX1	213985913	rs340874	T	C	T	1065	2206	185	880	376	1830	51	89	48	236	478	201	0.483	0.492	0.481	1103
1	LYPLAL1	219527177	rs2785980	T	C	C	717	2202	107	610	376	1826	96	77	15	396	424	93	0.326	0.285	0.334	1101
1	MTR	236885200	rs1805087	A	G	G	397	2204	54	343	378	1826	137	50	2	600	283	30	0.180	0.143	0.188	1102
2	DPYSL5	26930006	rs1371614	C	T	T	572	2204	111	461	378	1826	87	93	9	521	323	69	0.260	0.294	0.252	1102
2	GCKR	27518370	rs780094	T	C	T	1043	2208	153	890	378	1830	69	87	33	251	438	226	0.472	0.405	0.486	1104
2	MAP3K19	134998059	rs1530559	A	G	A	765	2202	139	626	374	1828	73	89	25	391	420	103	0.347	0.372	0.342	1101
2	RBMS1	160460949	rs6742799	A	C	C	389	2200	62	327	374	1826	130	52	5	618	263	32	0.177	0.166	0.179	1100
2	FIGN	163641436	rs2119289	C	G	C	284	2204	44	240	378	1826	149	36	4	686	214	13	0.129	0.116	0.131	1102
2	COBLL1	164694691	rs7607980	T	C	C	318	2204	41	277	378	1826	151	35	3	655	239	19	0.144	0.108	0.152	1102
2	G6PC2	168906638	rs560887	T	C	T	581	2208	88	493	378	1830	114	62	13	491	355	69	0.263	0.233	0.269	1104
2	G6PC2	168917561	rs563694	C	A	C	679	2194	110	569	378	1816	94	80	15	419	409	80	0.309	0.291	0.313	1097
2	IRS1	226203364	rs2943634	A	C	A	673	2176	112	561	374	1802	88	86	13	424	393	84	0.309	0.299	0.311	1088
2	IRS1	226795828	rs1801278	C	T	T	188	2206	39	149	376	1830	151	35	2	770	141	4	0.085	0.104	0.081	1103
3	PPARG	12348985	rs17036328	T	C	C	205	2208	31	174	378	1830	161	25	3	752	152	11	0.093	0.082	0.095	1104
3	PPARG	12351626	rs1801282	C	G	G	194	2208	29	165	378	1830	163	23	3	759	147	9	0.088	0.077	0.090	1104
3	UBE2E2	23413299	rs1496653	A	G	G	394	2208	62	332	378	1830	135	46	8	611	276	28	0.178	0.164	0.181	1104
3	AMT	49417897	rs11715915	C	T	T	713	2188	128	585	376	1812	83	82	23	422	383	101	0.326	0.340	0.323	1094
3	ADCY5	123346931	rs11708067	A	G	G	359	2192	70	289	376	1816	124	58	6	636	255	17	0.164	0.186	0.159	1096
3	SLC2A2	170999732	rs11920090	T	A	A	352	2204	57	295	376	1828	136	47	5	644	245	25	0.160	0.152	0.161	1102
3	IGF2BP2	185793899	rs4402960	G	T	T	695	2208	148	547	378	1830	68	94	27	441	401	73	0.315	0.392	0.299	1104
3	IGF2BP2	185795604	rs7651090	A	G	G	696	2208	147	549	378	1830	67	97	25	436	409	70	0.315	0.389	0.300	1104
3	ADIPOQ	186853103	rs2241766	T	G	G	400	2206	70	330	378	1828	126	56	7	608	282	24	0.181	0.185	0.181	1103
4	WFS1	6288259	rs4458523	T	G	T	825	2194	140	685	372	1822	73	86	27	361	415	135	0.376	0.376	0.376	1097
4	FAM13A	88820118	rs3822072	G	A	A	1065	2196	189	876	374	1822	43	99	45	230	486	195	0.485	0.505	0.481	1098
4	TET2	105160479	rs9884482	T	C	C	870	2196	142	728	374	1822	75	82	30	319	456	136	0.396	0.380	0.400	1098
4	PDGFC	156798972	rs4691380	C	T	T	827	2204	145	682	376	1828	69	93	26	353	440	121	0.375	0.386	0.373	1102
5	IRX1	4355595	rs17727202	T	C	C	165	2208	27	138	378	1830	162	27	0	779	134	2	0.075	0.071	0.075	1104
5	ANKRD55	56510924	rs459193	A	G	A	663	2208	105	558	378	1830	100	73	16	451	370	94	0.300	0.278	0.305	1104
5	ZBED3	77130042	rs7708285	G	A	G	658	2208	128	530	378	1830	84	82	23	460	380	75	0.298	0.339	0.290	1104
5	PCSK1	96207022	rs13179048	C	A	A	623	2208	99	524	378	1830	102	75	12	454	398	63	0.282	0.262	0.286	1104
5	PCSK1	96295001	rs17085593	C	G	G	637	2204	103	534	376	1828	99	75	14	443	408	63	0.289	0.274	0.292	1102
5	PCSK1	96393194	rs6235	C	G	G	565	2200	88	477	374	1826	107	72	8	481	387	45	0.257	0.235	0.261	1100
6	RRB1	7212967	rs17762454	C	T	T	615	2188	95	520	372	1816	102	73	11	462	372	74	0.281	0.255	0.286	1094
6	RREB1	7231610	rs9379084	G	A	A	332	2208	66	266	378	1830	130	52	7	668	228	19	0.150	0.175	0.145	1104
6	CDKAL1	20679478	rs7756992	A	G	G	549	2192	86	463	374	1818	108	72	7	503	349	57	0.250	0.230	0.255	1096
6	CDKAL1	20686765	rs9368222	C	A	A	522	2206	80	442	378	1828	115	68	6	523	340	51	0.237	0.212	0.242	1103
6	RSPO3	127131790	rs2745353	C	T	T	1075	2206	177	898	378	1828	52	97	40	240	450	224	0.487	0.468	0.491	1103
7	DGKB	15024684	rs2191349	G	T	G	987	2202	175	812	376	1826	56	89	43	286	442	185	0.448	0.465	0.445	1101
7	GCK	44189469	rs1799884	C	T	T	424	2208	77	347	378	1830	119	63	7	599	285	31	0.192	0.204	0.190	1104
7	GCK	44196069	rs4607517	G	A	A	409	2184	74	335	378	1806	120	64	5	597	277	29	0.187	0.196	0.185	1092
7	GRB10	50690548	rs933360	C	T	C	530	2196	91	439	374	1822	108	67	12	523	337	51	0.241	0.243	0.241	1098
7	GRB10	50723882	rs6943153	T	C	T	602	2184	102	500	372	1812	98	74	14	469	374	63	0.276	0.274	0.276	1092
7	HIP1	75546898	rs1167800	A	G	G	974	2208	156	818	378	1830	62	98	29	281	450	184	0.441	0.413	0.447	1104
8	PPP1R3B	9326086	rs4841132	A	G	A	135	2208	22	113	378	1830	167	22	0	809	99	7	0.061	0.058	0.062	1104
8	PPP1R3B	9330085	rs7004769	A	G	A	407	2204	63	344	378	1826	131	53	5	609	264	40	0.185	0.167	0.188	1102
8	ANK1	41651740	rs12549902	G	A	G	1025	2198	175	850	378	1820	54	95	40	238	494	178	0.466	0.463	0.467	1099
8	SLC30A8	117172544	rs13266634	C	T	T	581	2208	98	483	378	1830	104	72	13	485	377	53	0.263	0.259	0.264	1104
8	SLC30A8	117172786	rs3802177	G	A	A	567	2208	95	472	378	1830	106	71	12	490	378	47	0.257	0.251	0.258	1104
8	SLC30A8	117173494	rs11558471	A	G	G	601	2206	99	502	378	1828	103	73	13	466	394	54	0.272	0.262	0.275	1103
9	GLIS3	4287466	rs7041847	A	G	G	1034	2202	167	867	376	1826	63	83	42	245	469	199	0.470	0.444	0.475	1101
9	GLIS3	4289050	rs7034200	C	A	C	1092	2206	180	912	378	1828	60	78	51	225	466	223	0.495	0.476	0.499	1103
9	GLIS3	4293150	rs10814916	A	C	A	1043	2194	171	872	378	1816	63	81	45	234	476	198	0.475	0.452	0.480	1097
9	CDKN2B	22134095	rs10811661	T	C	C	423	2188	73	350	374	1814	122	57	8	592	280	35	0.193	0.195	0.193	1094
9	SARDH	133734024	rs573904	C	T	T	626	2206	120	506	378	1828	85	88	16	479	364	71	0.284	0.317	0.277	1103
10	CDC123	12265895	rs11257655	C	T	T	503	2208	93	410	378	1830	107	71	11	545	330	40	0.228	0.246	0.224	1104
10	CDC123	12286011	rs12779790	A	G	G	433	2208	78	355	378	1830	120	60	9	587	301	27	0.196	0.206	0.194	1104
10	CUBN	17114152	rs1801222	A	G	A	607	2198	114	493	376	1822	90	82	16	488	353	70	0.276	0.303	0.271	1099
10	HKDC1	69223185	rs4746822	C	T	C	968	2204	161	807	378	1826	60	97	32	276	467	170	0.439	0.426	0.442	1102
10	HHEX	92722319	rs7923866	C	T	T	783	2206	132	651	378	1828	75	96	18	374	429	111	0.355	0.349	0.356	1103
10	ADRA2A	111282335	rs10885122	T	G	T	292	2206	48	244	376	1830	144	40	4	687	212	16	0.132	0.128	0.133	1103
10	TCF7L2	112994312	rs34872471	T	C	C	759	2208	140	619	378	1830	72	94	23	394	423	98	0.344	0.370	0.338	1104
10	TCF7L2	112996282	rs4506565	A	T	T	819	2204	147	672	378	1826	68	95	26	356	442	115	0.372	0.389	0.368	1102
10	TCF7L2	112998590	rs7903146	C	T	T	774	2206	144	630	378	1828	73	88	28	387	424	103	0.351	0.381	0.345	1103
11	DUSP8	1675619	rs2334499	C	T	T	967	2180	172	795	376	1804	50	104	34	279	451	172	0.444	0.457	0.441	1090
11	KCNJ11	17387083	rs5215	C	T	C	772	2200	146	626	376	1824	66	98	24	396	406	110	0.351	0.388	0.343	1100
11	CRY2	45851540	rs11605924	A	C	C	1096	2208	178	918	378	1830	52	96	41	238	436	241	0.496	0.471	0.502	1104
11	MADD	47314769	rs7944584	A	T	T	711	2204	110	601	378	1826	92	84	13	412	401	100	0.323	0.291	0.329	1102
11	OR4S1	48311808	rs1483121	G	A	A	340	2204	53	287	378	1826	139	47	3	640	259	14	0.154	0.140	0.157	1102
11	FADS1	61804006	rs174550	T	C	C	675	2196	118	557	378	1818	88	84	17	432	397	80	0.307	0.312	0.306	1098
11	ARAP1	72721940	rs11603334	G	A	A	283	2208	43	240	378	1830	149	37	3	691	208	16	0.128	0.114	0.131	1104
11	MTNR1B	92940662	rs1387153	C	T	T	646	2206	136	510	378	1828	75	92	22	470	378	66	0.293	0.360	0.279	1103
11	MTNR1B	92965261	rs10830962	C	G	G	935	2196	180	755	374	1822	47	100	40	310	447	154	0.426	0.481	0.414	1098
11	MTNR1B	92975544	rs10830963	C	G	G	607	2204	132	475	378	1826	78	90	21	504	343	66	0.275	0.349	0.260	1102
12	GLS2	56471554	rs2657879	A	G	G	473	2206	83	390	378	1828	113	69	7	568	302	44	0.214	0.220	0.213	1103
12	IGF1	102481791	rs35767	A	G	A	346	2202	54	292	378	1824	139	46	4	650	232	30	0.157	0.143	0.160	1101
12	HNF1A	121022883	rs7957197	T	A	A	464	2200	71	393	376	1824	125	55	8	560	311	41	0.211	0.189	0.215	1100
12	P2RX2	132465032	rs10747083	G	A	G	769	2206	130	639	378	1828	87	74	28	373	443	98	0.349	0.344	0.350	1103
13	PDX1	27917061	rs2293941	G	A	A	534	2204	103	431	378	1826	97	81	11	538	319	56	0.242	0.272	0.236	1102
13	KL	32980164	rs576674	G	A	G	504	2192	83	421	376	1816	112	69	7	524	347	37	0.230	0.221	0.232	1096
14	WARS	100372924	rs3783347	G	T	T	383	2208	53	330	378	1830	141	43	5	609	282	24	0.173	0.140	0.180	1104
15	C2CD4A	62090956	rs4502156	T	C	C	1011	2202	174	837	378	1824	57	90	42	264	459	189	0.459	0.460	0.459	1101
15	C2CD4B	62141763	rs11071657	A	G	G	875	2208	156	719	378	1830	61	100	28	328	455	132	0.396	0.413	0.393	1104
16	FTO	53767042	rs1421085	T	C	C	914	2204	149	765	376	1828	65	97	26	303	457	154	0.415	0.396	0.418	1102
16	FTO	53782363	rs8050136	C	A	A	896	2194	154	742	376	1818	66	90	32	317	442	150	0.408	0.410	0.408	1097
16	CTRB2	75211105	rs9921586	G	T	T	281	2208	47	234	378	1830	143	45	1	693	210	12	0.127	0.124	0.128	1104
17	GLP2R	9888058	rs17676067	T	C	C	598	2206	120	478	376	1830	90	76	22	498	356	61	0.271	0.319	0.261	1103
17	HNF1B	37738049	rs4430796	A	G	A	1009	2206	169	840	378	1828	63	83	43	269	450	195	0.457	0.447	0.460	1103
19	CILP2	19547663	rs16996148	G	T	T	171	2208	26	145	378	1830	163	26	0	774	137	4	0.077	0.069	0.079	1104
19	PEPD	33408159	rs731839	G	A	G	762	2196	136	626	376	1820	75	90	23	383	428	99	0.347	0.362	0.344	1098
19	GIPR	45693376	rs2302593	C	G	G	1082	2198	188	894	378	1820	43	104	42	235	456	219	0.492	0.497	0.491	1099
20	FOXA2	22578963	rs6048205	A	G	G	110	2208	10	100	378	1830	179	10	0	819	92	4	0.050	0.026	0.055	1104
20	TOP1	41115265	rs6072275	G	A	A	336	2206	54	282	378	1828	138	48	3	654	238	22	0.152	0.143	0.154	1103
20	ZHX3	41203988	rs17265513	T	C	C	406	2204	68	338	378	1826	127	56	6	609	270	34	0.184	0.180	0.185	1102
20	SLC17A9	62967547	rs3746750	A	G	A	759	2200	111	648	376	1824	94	77	17	362	452	98	0.345	0.295	0.355	1100
21	BACE2	41209710	rs737288	G	T	T	773	2188	130	643	374	1814	74	96	17	373	425	109	0.353	0.348	0.354	1094
21	BACE2	41211811	rs6517656	G	A	A	458	2208	78	380	378	1830	118	64	7	573	304	38	0.207	0.206	0.208	1104

Main characteristics of the variants for the Caucasian (CAU) ethnicity.

CHROM, Chromosome code; LOCUS, Locus/Gene; POS, Base-pair coordinate [GRCh38]; ID, Variant ID; REF, Reference allele; ALT, Alternate allele; A1, Counted allele in logistic regression; A1_CT, Total A1 allele count; ALLELE_CT, Allele observation count; A1_CASE_CT, A1 count in cases; A1_CTRL_CT, A1 count in controls; CASE_ALLELE_CT, Case allele observation count; CTRL_ALLELE_CT, Control allele observation count; CASE_NON_A1_CT, Case genotypes with 0 copies of A1; CASE_HET_A1_CT, Case genotypes with 1 copy of A1; CASE_HOM_A1_CT, Case genotypes with 2 copies of A1; CTRL_NON_A1_CT, Control genotypes with 0 copies of A1; CTRL_HET_A1_CT, Control genotypes with 1 copy of A1; CTRL_HOM_A1_CT, Control genotypes with 2 copies of A1; A1_FREQ, A1 allele frequency; A1_CASE_FREQ, A1 allele frequency in cases; A1_CTRL_FREQ, A1 allele frequency in controls; OBS_CT, Number of samples in the regression.

**Table 2:** HIS Characteristics of variants. HISPANIC.

CHROM	LOCUS	POS	ID	REF	ALT	A1	A1_CT	ALLELE_CT	A1_CASE_CT	A1_CTRL_CT	CASE_ALLELE_CT	CTRL_ALLELE_CT	CASE_NON_A1_CT	CASE_HET_A1_CT	CASE_HOM_A1_CT	CTRL_NON_A1_CT	CTRL_HET_A1_CT	CTRL_HOM_A1_CT	A1_FREQ	A1_CASE_FREQ	A1_CTRL_FREQ	OBS_CT
1	MTHFR	11794419	rs1801131	T	G	G	137	930	25	112	166	764	59	23	1	279	94	9	0.147	0.151	0.147	465
1	MTHFR	11796321	rs1801133	G	A	A	373	936	65	308	166	770	30	41	12	139	184	62	0.399	0.392	0.400	468
1	PROX1	213985913	rs340874	T	C	C	330	936	50	280	166	770	44	28	11	155	180	50	0.353	0.301	0.364	468
1	LYPLAL1-AS1	219527177	rs2785980	T	C	T	406	936	78	328	166	770	29	30	24	145	152	88	0.434	0.470	0.426	468
1	MTR	236885200	rs1805087	A	G	G	195	938	33	162	166	772	54	25	4	240	130	16	0.208	0.199	0.210	469
2	DPYSL5	26930006	rs1371614	C	T	T	396	934	80	316	164	770	21	42	19	145	164	76	0.424	0.488	0.410	467
2	GCKR	27518370	rs780094	T	C	T	308	930	48	260	164	766	44	28	10	164	178	41	0.331	0.293	0.339	465
2	MAP3K19	134998059	rs1530559	A	G	A	308	934	49	259	164	770	41	33	8	174	163	48	0.330	0.299	0.336	467
2	RBMS1	160460949	rs6742799	A	C	C	130	928	22	108	166	762	62	20	1	279	96	6	0.140	0.133	0.142	464
2	FIGN	163641436	rs2119289	C	G	C	99	938	22	77	166	772	61	22	0	311	73	2	0.106	0.133	0.100	469
2	COBLL1	164694691	rs7607980	T	C	C	65	938	12	53	166	772	71	12	0	335	49	2	0.069	0.072	0.069	469
2	G6PC2	168906638	rs560887	T	C	T	94	938	12	82	166	772	72	10	1	307	76	3	0.100	0.072	0.106	469
2	G6PC2	168917561	rs563694	C	A	C	119	938	15	104	166	772	70	11	2	286	96	4	0.127	0.090	0.135	469
2	IRS1	226203364	rs2943634	A	C	A	190	932	25	165	164	768	61	17	4	247	109	28	0.204	0.152	0.215	466
2	IRS1	226795828	rs1801278	C	T	T	60	938	8	52	166	772	75	8	0	338	44	4	0.064	0.048	0.067	469
3	PPARG	12348985	rs17036328	T	C	C	146	938	21	125	166	772	64	17	2	273	101	12	0.156	0.127	0.162	469
3	PPARG	12351626	rs1801282	C	G	G	123	938	17	106	166	772	67	15	1	291	84	11	0.131	0.102	0.137	469
3	UBE2E2	23413299	rs1496653	A	G	G	107	938	13	94	166	772	71	11	1	299	80	7	0.114	0.078	0.122	469
3	AMT	49417897	rs11715915	C	T	T	140	938	30	110	166	772	58	20	5	289	84	13	0.149	0.181	0.142	469
3	ADCY5	123346931	rs11708067	A	G	G	335	936	54	281	166	770	39	34	10	154	181	50	0.358	0.325	0.365	468
3	SLC2A2	170999732	rs11920090	T	A	A	129	938	19	110	166	772	64	19	0	288	86	12	0.138	0.114	0.142	469
3	IGF2BP2	185793899	rs4402960	G	T	T	237	938	51	186	166	772	41	33	9	222	142	22	0.253	0.307	0.241	469
3	IGF2BP2	185795604	rs7651090	A	G	G	232	934	50	182	166	768	41	34	8	221	144	19	0.248	0.301	0.237	467
3	ADIPOQ	186853103	rs2241766	T	G	G	168	938	32	136	166	772	54	26	3	259	118	9	0.179	0.193	0.176	469
4	WFS1	6288259	rs4458523	T	G	T	292	926	46	246	162	764	38	40	3	174	170	38	0.315	0.284	0.322	463
4	FAM13A	88820118	rs3822072	G	A	A	401	934	72	329	166	768	26	42	15	121	197	66	0.429	0.434	0.428	467
4	TET2	105160479	rs9884482	T	C	C	398	934	62	336	166	768	33	38	12	123	186	75	0.426	0.373	0.438	467
4	PDGFC	156798972	rs4691380	C	T	T	325	932	64	261	166	766	34	34	15	171	163	49	0.349	0.386	0.341	466
5	IRX1	4355595	rs17727202	T	C	C	40	938	5	35	166	772	78	5	0	351	35	0	0.043	0.030	0.045	469
5	ANKRD55	56510924	rs459193	A	G	A	219	934	47	172	166	768	46	27	10	235	126	23	0.234	0.283	0.224	467
5	ZBED3	77130042	rs7708285	G	A	G	338	938	62	276	166	772	30	44	9	164	168	54	0.360	0.373	0.358	469
5	PCSK1	96207022	rs13179048	C	A	A	173	936	25	148	166	770	59	23	1	253	116	16	0.185	0.151	0.192	468
5	PCSK1	96295001	rs17085593	C	G	G	182	938	27	155	166	772	57	25	1	248	121	17	0.194	0.163	0.201	469
5	PCSK1	96393194	rs6235	C	G	G	182	938	27	155	166	772	56	27	0	251	115	20	0.194	0.163	0.201	469
6	RRB1	7212967	rs17762454	C	T	T	360	936	70	290	166	770	29	38	16	148	184	53	0.385	0.422	0.377	468
6	RREB1	7231610	rs9379084	G	A	A	51	938	7	44	166	772	76	7	0	344	40	2	0.054	0.042	0.057	469
6	CDKAL1	20679478	rs7756992	A	G	G	288	934	57	231	166	768	36	37	10	190	157	37	0.308	0.343	0.301	467
6	CDKAL1	20686765	rs9368222	C	A	A	212	938	48	164	166	772	40	38	5	241	126	19	0.226	0.289	0.212	469
6	RSPO3	127131790	rs2745353	C	T	C	376	938	60	316	166	772	35	36	12	125	206	55	0.401	0.361	0.409	469
7	DGKB	15024684	rs2191349	G	T	T	384	936	78	306	166	770	20	48	15	132	200	53	0.410	0.470	0.397	468
7	GCK	44189469	rs1799884	C	T	T	180	936	38	142	166	770	51	26	6	258	112	15	0.192	0.229	0.184	468
7	GCK	44196069	rs4607517	G	A	A	168	928	32	136	162	766	54	22	5	261	108	14	0.181	0.198	0.178	464
7	GRB10	50690548	rs933360	C	T	C	341	936	72	269	166	770	29	36	18	166	169	50	0.364	0.434	0.349	468
7	GRB10	50723882	rs6943153	T	C	C	460	932	76	384	166	766	24	42	17	94	194	95	0.494	0.458	0.501	466
7	HIP1	75546898	rs1167800	A	G	G	285	938	50	235	166	772	42	32	9	186	165	35	0.304	0.301	0.304	469
8	PPP1R3B	9326086	rs4841132	A	G	A	226	936	39	187	164	772	49	27	6	219	147	20	0.241	0.238	0.242	468
8	PPP1R3B	9330085	rs7004769	A	G	A	367	938	63	304	166	772	30	43	10	135	198	53	0.391	0.380	0.394	469
8	ANK1	41651740	rs12549902	G	A	G	387	932	72	315	164	768	27	38	17	124	205	55	0.415	0.439	0.410	466
8	SLC30A8	117172544	rs13266634	C	T	T	235	936	43	192	166	770	48	27	8	219	140	26	0.251	0.259	0.249	468
8	SLC30A8	117172786	rs3802177	G	A	A	232	938	41	191	166	772	49	27	7	222	137	27	0.247	0.247	0.247	469
8	SLC30A8	117173494	rs11558471	A	G	G	244	938	44	200	166	772	47	28	8	216	140	30	0.260	0.265	0.259	469
9	GLIS3	4287466	rs7041847	A	G	G	394	936	57	337	166	770	37	35	11	121	191	73	0.421	0.343	0.438	468
9	GLIS3	4289050	rs7034200	C	A	C	461	936	71	390	166	770	27	41	15	93	194	98	0.493	0.428	0.506	468
9	GLIS3	4293150	rs10814916	A	C	A	433	932	66	367	166	766	28	44	11	105	189	89	0.465	0.398	0.479	466
9	CDKN2B	22134095	rs10811661	T	C	C	119	934	24	95	166	768	60	22	1	295	83	6	0.127	0.145	0.124	467
9	SARDH	133734024	rs573904	C	T	T	201	934	36	165	166	768	51	28	4	234	135	15	0.215	0.217	0.215	467
10	CDC123	12265895	rs11257655	C	T	T	245	938	46	199	166	772	44	32	7	213	147	26	0.261	0.277	0.258	469
10	CDC123	12286011	rs12779790	A	G	G	135	936	23	112	166	770	61	21	1	282	94	9	0.144	0.139	0.145	468
10	CUBN	17114152	rs1801222	A	G	A	245	936	39	206	166	770	51	25	7	200	164	21	0.262	0.235	0.268	468
10	HKDC1	69223185	rs4746822	C	T	T	457	936	84	373	166	770	22	38	23	105	187	93	0.488	0.506	0.484	468
10	HHEX	92722319	rs7923866	C	T	C	466	936	90	376	166	770	17	42	24	104	186	95	0.498	0.542	0.488	468
10	ADRA2A	111282335	rs10885122	T	G	T	172	938	37	135	166	772	54	21	8	262	113	11	0.183	0.223	0.175	469
10	TCF7L2	112994312	rs34872471	T	C	C	192	938	29	163	166	772	58	21	4	240	129	17	0.205	0.175	0.211	469
10	TCF7L2	112996282	rs4506565	A	T	T	215	936	38	177	166	770	52	24	7	228	137	20	0.230	0.229	0.230	468
10	TCF7L2	112998590	rs7903146	C	T	T	190	936	30	160	166	770	57	22	4	240	130	15	0.203	0.181	0.208	468
11	DUSP8	1675619	rs2334499	C	T	T	425	934	85	340	166	768	23	35	25	123	182	79	0.455	0.512	0.443	467
11	KCNJ11	17387083	rs5215	C	T	C	294	932	55	239	166	766	36	39	8	185	157	41	0.315	0.331	0.312	466
11	CRY2	45851540	rs11605924	A	C	C	468	938	72	396	166	772	23	48	12	95	186	105	0.499	0.434	0.513	469
11	MADD	47314769	rs7944584	A	T	T	138	938	24	114	166	772	60	22	1	283	92	11	0.147	0.145	0.148	469
11	OR4S1	48311808	rs1483121	G	A	A	57	938	14	43	166	772	70	12	1	344	41	1	0.061	0.084	0.056	469
11	FADS1	61804006	rs174550	T	C	T	359	928	62	297	164	764	37	28	17	160	147	75	0.387	0.378	0.389	464
11	ARAP1	72721940	rs11603334	G	A	A	71	936	8	63	166	770	76	6	1	326	55	4	0.076	0.048	0.082	468
11	MTNR1B	92940662	rs1387153	C	T	T	163	936	38	125	166	770	50	28	5	270	105	10	0.174	0.229	0.162	468
11	MTNR1B	92965261	rs10830962	C	G	G	309	938	62	247	166	772	34	36	13	182	161	43	0.329	0.373	0.320	469
11	MTNR1B	92975544	rs10830963	C	G	G	124	938	27	97	166	772	56	27	0	294	87	5	0.132	0.163	0.126	469
12	GLS2	56471554	rs2657879	A	G	G	234	938	34	200	166	772	53	26	4	212	148	26	0.249	0.205	0.259	469
12	IGF1	102481791	rs35767	A	G	A	239	936	50	189	166	770	39	38	6	219	143	23	0.255	0.301	0.245	468
12	HNF1A	121022883	rs7957197	T	A	A	106	938	18	88	166	772	66	16	1	310	64	12	0.113	0.108	0.114	469
12	P2RX2	132465032	rs10747083	G	A	G	243	938	37	206	166	772	52	25	6	201	164	21	0.259	0.223	0.267	469
13	PDX1	27917061	rs2293941	G	A	A	277	938	53	224	166	772	35	43	5	206	136	44	0.295	0.319	0.290	469
13	KL	32980164	rs576674	G	A	G	344	934	60	284	164	770	31	42	9	161	164	60	0.368	0.366	0.369	467
14	WARS	100372924	rs3783347	G	T	T	96	938	14	82	166	772	69	14	0	308	74	4	0.102	0.084	0.106	469
15	C2CD4A	62090956	rs4502156	T	C	T	289	936	50	239	166	770	43	30	10	184	163	38	0.309	0.301	0.310	468
15	C2CD4B	62141763	rs11071657	A	G	A	393	936	65	328	166	770	34	33	16	138	166	81	0.420	0.392	0.426	468
16	FTO	53767042	rs1421085	T	C	C	151	938	22	129	166	772	61	22	0	267	109	10	0.161	0.133	0.167	469
16	FTO	53782363	rs8050136	C	A	A	195	938	31	164	166	772	54	27	2	240	128	18	0.208	0.187	0.212	469
16	CTRB2	75211105	rs9921586	G	T	T	118	938	19	99	166	772	65	17	1	298	77	11	0.126	0.114	0.128	469
17	GLP2R	9888058	rs17676067	T	C	C	109	936	23	86	166	770	62	19	2	301	82	2	0.116	0.139	0.112	468
17	HNF1B	37738049	rs4430796	A	G	G	322	938	66	256	166	772	32	36	15	181	154	51	0.343	0.398	0.332	469
19	CILP2	19547663	rs16996148	G	T	T	57	938	13	44	166	772	71	11	1	343	42	1	0.061	0.078	0.057	469
19	PEPD	33408159	rs731839	G	A	G	416	934	78	338	164	770	22	42	18	118	196	71	0.445	0.476	0.439	467
19	GIPR	45693376	rs2302593	C	G	C	387	934	83	304	166	768	21	41	21	146	172	66	0.414	0.500	0.396	467
20	FOXA2	22578963	rs6048205	A	G	G	50	938	12	38	166	772	72	10	1	352	30	4	0.053	0.072	0.049	469
20	TOP1	41115265	rs6072275	G	A	A	118	938	20	98	166	772	65	16	2	293	88	5	0.126	0.120	0.127	469
20	ZHX3	41203988	rs17265513	T	C	C	72	938	15	57	166	772	69	13	1	330	55	1	0.077	0.090	0.074	469
20	SLC17A9	62967547	rs3746750	A	G	A	314	934	63	251	164	770	30	41	11	167	185	33	0.336	0.384	0.326	467
21	BACE2	41209710	rs737288	G	T	T	194	934	39	155	164	770	52	21	9	246	123	16	0.208	0.238	0.201	467
21	BACE2	41211811	rs6517656	G	A	A	167	936	36	131	166	770	55	20	8	270	99	16	0.178	0.217	0.170	468

Main characteristics of the variants for the Hispanic (HIS) ethnicity.

CHROM, Chromosome code; LOCUS, Locus/Gene; POS, Base-pair coordinate [GRCh38]; ID, Variant ID; REF, Reference allele; ALT, Alternate allele; A1, Counted allele in logistic regression; A1_CT, Total A1 allele count; ALLELE_CT, Allele observation count; A1_CASE_CT, A1 count in cases; A1_CTRL_CT, A1 count in controls; CASE_ALLELE_CT, Case allele observation count; CTRL_ALLELE_CT, Control allele observation count; CASE_NON_A1_CT, Case genotypes with 0 copies of A1; CASE_HET_A1_CT, Case genotypes with 1 copy of A1; CASE_HOM_A1_CT, Case genotypes with 2 copies of A1; CTRL_NON_A1_CT, Control genotypes with 0 copies of A1; CTRL_HET_A1_CT, Control genotypes with 1 copy of A1; CTRL_HOM_A1_CT, Control genotypes with 2 copies of A1; A1_FREQ, A1 allele frequency; A1_CASE_FREQ, A1 allele frequency in cases; A1_CTRL_FREQ, A1 allele frequency in controls; OBS_CT, Number of samples in the regression.

**Supplementary Tables 2** CAU-2HIS show, respectively, for each ethnicity, logistic regression analysis performed for the 98 SNPs and 1573 samples. **Tables 3a** CAU and **3b** HIS extract, respectively, the main relevant findings for the two ethnic strata considered; specifically, these tables show the SNPs for which a discovery (*p*-value ≤0.05, or *q*-value ≤ 0.05, or *lfdr* ≤ 0.1) was obtained in at least one of the SNP genetic tests performed.

**Table 3:** CAU (SNP + GROUP) MODELS. SIGNIFICANT SNPs. CAUCASIAN.

									ADDITIVE				DOMINANT				RECESSIVE				HETHOM							
									ADD				DOM				REC				HOM				HET			
CHROM	LOCUS	POS	ID	REF	ALT	A1	A1_FREQ	OBS_CT	OR_CI95	pvalue	qvalue	lfdr	OR_CI95	pvalue	qvalue	lfdr	OR_CI95	pvalue	qvalue	lfdr	OR_CI95	pvalue	qvalue	lfdr	OR_CI95	pvalue	qvalue	lfdr
1	LYPLAL1	219527177	rs2785980	T	C	C	0.326	1101	0.79 (0.62-1.01)	0.062	0.037	0.224	0.74 (0.54-1.02)	0.064	0.036	0.231	0.74 (0.42-1.31)	0.308	0.219	0.997	0.65 (0.36-1.18)	0.161	0.196	0.865	0.76 (0.55-1.06)	0.110	0.141	0.714
1	MTR	236885200	rs1805087	A	G	G	0.180	1102	0.73 (0.53-1.00)	0.050	0.030	0.169	0.75 (0.53-1.06)	0.098	0.054	0.383	0.31 (0.08-1.22)	0.095	0.081	0.512	0.29 (0.07-1.24)	0.096	0.125	0.645	0.79 (0.56-1.13)	0.205	0.235	0.926
2	DPYSL5	26930006	rs1371614	C	T	T	0.260	1102	1.21 (0.95-1.54)	0.132	0.076	0.488	1.53 (1.12-2.10)	0.008	0.006	0.020	0.59 (0.29-1.20)	0.145	0.118	0.744	0.75 (0.36-1.57)	0.448	0.385	0.981	1.70 (1.23-2.35)	0.001	0.015	0.014
2	GCKR	27518370	rs780094	T	C	T	0.472	1104	0.74 (0.59-0.92)	0.007	0.006	0.019	0.67 (0.48-0.93)	0.016	0.010	0.042	0.66 (0.44-0.99)	0.042	0.037	0.196	0.54 (0.35-0.86)	0.009	0.015	0.051	0.73 (0.51-1.04)	0.080	0.107	0.549
2	COBLL1	164694691	rs7607980	T	C	C	0.144	1102	0.67 (0.47-0.95)	0.023	0.014	0.066	0.63 (0.43-0.92)	0.016	0.010	0.041	0.73 (0.24-2.28)	0.594	0.344	1.000	0.66 (0.20-2.15)	0.489	0.406	0.981	0.62 (0.42-0.93)	0.020	0.029	0.117
3	IGF2BP2	185793899	rs4402960	G	T	T	0.315	1104	1.54 (1.21-1.95)	0.000	0.006	0.004	1.66 (1.20-2.30)	0.002	0.006	0.008	1.89 (1.18-3.04)	0.008	0.008	0.033	2.37 (1.42-3.95)	0.001	0.015	0.012	1.53 (1.09-2.15)	0.015	0.022	0.085
3	IGF2BP2	185795604	rs7651090	A	G	G	0.315	1104	1.52 (1.20-1.94)	0.001	0.006	0.004	1.67 (1.20-2.31)	0.002	0.006	0.008	1.79 (1.10-2.92)	0.020	0.018	0.078	2.27 (1.34-3.85)	0.002	0.015	0.019	1.56 (1.11-2.19)	0.011	0.016	0.062
5	ZBED3	77130042	rs7708285	G	A	G	0.298	1104	1.24 (0.98-1.57)	0.078	0.046	0.292	1.24 (0.90-1.70)	0.180	0.097	0.623	1.52 (0.92-2.50)	0.099	0.084	0.536	1.63 (0.97-2.76)	0.066	0.090	0.460	1.16 (0.83-1.62)	0.376	0.361	0.979
9	GLIS3	4287466	rs7041847	A	G	G	0.470	1101	0.87 (0.69-1.09)	0.228	0.119	0.663	0.71 (0.51-1.00)	0.048	0.028	0.156	1.02 (0.70-1.49)	0.924	0.444	1.000	0.80 (0.52-1.23)	0.311	0.316	0.973	0.67 (0.47-0.97)	0.033	0.047	0.210
9	GLIS3	4289050	rs7034200	C	A	C	0.495	1103	0.90 (0.72-1.13)	0.363	0.169	0.769	0.68 (0.49-0.96)	0.030	0.018	0.086	1.14 (0.80-1.62)	0.485	0.300	1.000	0.84 (0.55-1.27)	0.401	0.368	0.980	0.61 (0.42-0.89)	0.010	0.016	0.059
9	GLIS3	4293150	rs10814916	A	C	A	0.475	1097	0.87 (0.70-1.10)	0.244	0.126	0.681	0.67 (0.48-0.94)	0.021	0.013	0.055	1.10 (0.76-1.59)	0.621	0.346	1.000	0.81 (0.53-1.24)	0.335	0.331	0.976	0.61 (0.43-0.89)	0.009	0.015	0.053
9	SARDH	133734024	rs573904	C	T	T	0.284	1103	1.20 (0.94-1.53)	0.140	0.081	0.512	1.32 (0.96-1.81)	0.083	0.047	0.318	1.08 (0.62-1.90)	0.786	0.392	1.000	1.24 (0.69-2.24)	0.475	0.400	0.981	1.34 (0.96-1.86)	0.084	0.111	0.574
11	KCNJ11	17387083	rs5215	C	T	C	0.351	1100	1.24 (0.98-1.56)	0.071	0.042	0.262	1.45 (1.04-2.01)	0.027	0.016	0.073	1.09 (0.68-1.75)	0.722	0.372	1.000	1.35 (0.81-2.26)	0.251	0.282	0.956	1.48 (1.05-2.08)	0.026	0.038	0.158
11	MTNR1B	92940662	rs1387153	C	T	T	0.293	1103	1.49 (1.17-1.89)	0.001	0.006	0.006	1.63 (1.18-2.24)	0.003	0.006	0.009	1.71 (1.02-2.85)	0.040	0.036	0.185	2.12 (1.23-3.65)	0.007	0.015	0.041	1.54 (1.10-2.16)	0.011	0.017	0.065
11	MTNR1B	92965261	rs10830962	C	G	G	0.426	1098	1.31 (1.05-1.64)	0.019	0.012	0.053	1.55 (1.08-2.21)	0.017	0.010	0.042	1.31 (0.88-1.93)	0.182	0.144	0.854	1.69 (1.06-2.69)	0.028	0.040	0.172	1.50 (1.03-2.18)	0.036	0.050	0.232
11	MTNR1B	92975544	rs10830963	C	G	G	0.275	1102	1.51 (1.19-1.91)	0.001	0.006	0.005	1.73 (1.26-2.37)	0.001	0.006	0.004	1.60 (0.96-2.67)	0.072	0.063	0.378	2.04 (1.18-3.51)	0.010	0.016	0.060	1.67 (1.20-2.33)	0.003	0.015	0.021
13	PDX1	27917061	rs2293941	G	A	A	0.242	1102	1.20 (0.93-1.54)	0.154	0.086	0.545	1.36 (0.99-1.87)	0.055	0.031	0.187	0.90 (0.46-1.75)	0.750	0.382	1.000	1.04 (0.52-2.05)	0.920	0.542	0.982	1.42 (1.03-1.97)	0.035	0.049	0.222
14	WARS	100372924	rs3783347	G	T	T	0.173	1104	0.73 (0.53-1.01)	0.057	0.034	0.200	0.68 (0.47-0.97)	0.032	0.018	0.091	0.99 (0.37-2.63)	0.981	0.455	1.000	0.88 (0.33-2.36)	0.802	0.507	0.982	0.66 (0.45-0.95)	0.027	0.040	0.168
17	GLP2R	9888058	rs17676067	T	C	C	0.271	1103	1.30 (1.02-1.65)	0.035	0.022	0.110	1.27 (0.92-1.74)	0.140	0.077	0.533	1.80 (1.07-3.01)	0.027	0.024	0.111	1.92 (1.12-3.29)	0.018	0.027	0.107	1.16 (0.83-1.62)	0.396	0.368	0.980
20	FOXA2	22578963	rs6048205	A	G	G	0.050	1104	0.47 (0.24-0.90)	0.023	0.014	0.065	0.47 (0.24-0.91)	0.026	0.016	0.072	0.50 (0.02-13.38)	0.682	0.362	1.000	0.48 (0.02-12.67)	0.658	0.456	0.982	0.51 (0.26-0.99)	0.045	0.063	0.301
20	SLC17A9	62967547	rs3746750	A	G	A	0.345	1100	0.73 (0.57-0.94)	0.015	0.009	0.039	0.65 (0.47-0.89)	0.008	0.006	0.019	0.78 (0.45-1.34)	0.369	0.255	1.000	0.63 (0.36-1.11)	0.109	0.140	0.706	0.65 (0.47-0.91)	0.012	0.019	0.071

Table that summarizes the most relevant results of the analysis of SNPs + Group models in Caucasian (CAU) ethnicity. ADD, Additive model; DOM, dominant model; REC, recessive model; HETHOM, heterozygous-homozygous model; CHROM, Chromosome code; LOCUS, Locus/Gene; POS, Base-pair coordinate [GRCh38]; ID, Variant ID; REF, Reference allele; ALT, Alternate allele; A1, Counted allele in logistic regression; A1_FREQ, minor allele frequency; OBS_CT, Number of samples in the regression; OR_CI95, odds ratio with 95% confidence interval.

**Table 3:** HIS (SNP + GROUP) MODELS. SIGNIFICANT SNPs. HISPANIC.

									ADDITIVE					DOMINANT				RECESSIVE				HETHOM							
									ADD					DOM				REC				HOM				HET			
CHROM	LOCUS	POS	ID	REF	ALT	A1	A1_FREQ	OBS_CT	OR_CI95	pvalue	qvalue	lfdr		OR_CI95	pvalue	qvalue	lfdr	OR_CI95	pvalue	qvalue	lfdr	OR_CI95	pvalue	qvalue	lfdr	OR_CI95	pvalue	qvalue	lfdr
1	PROX1	213985913	rs340874	T	C	C	0.353	468	0.78 (0.54-1.12)	0.177	0.139	0.594		0.62 (0.38-1.00)	0.049	0.023	0.079	1.06 (0.52-2.15)	0.869	0.219	0.643	0.81 (0.39-1.70)	0.585	0.371	0.858	0.56 (0.33-0.95)	0.032	0.044	0.120
2	DPYSL5	26930006	rs1371614	C	T	T	0.424	467	1.36 (0.98-1.88)	0.069	0.064	0.229		1.77 (1.03-3.03)	0.039	0.019	0.060	1.28 (0.72-2.27)	0.407	0.129	0.521	1.79 (0.90-3.55)	0.096	0.108	0.368	1.76 (0.99-3.12)	0.054	0.069	0.202
2	GCKR	27518370	rs780094	T	C	T	0.331	465	0.80 (0.55-1.16)	0.241	0.172	0.745		0.63 (0.39-1.02)	0.063	0.029	0.107	1.20 (0.57-2.53)	0.627	0.175	0.605	0.93 (0.43-2.01)	0.852	0.443	0.858	0.57 (0.34-0.96)	0.033	0.045	0.125
2	G6PC2	168917561	rs563694	C	A	C	0.127	469	0.64 (0.36-1.14)	0.132	0.111	0.460		0.54 (0.29-1.00)	0.051	0.024	0.083	2.25 (0.55-9.11)	0.258	0.093	0.417	1.96 (0.35-11.06)	0.447	0.325	0.849	0.48 (0.24-0.95)	0.034	0.046	0.126
2	IRS1	226203364	rs2943634	A	C	A	0.204	466	0.65 (0.42-1.01)	0.053	0.051	0.168		0.57 (0.33-0.98)	0.041	0.020	0.064	0.61 (0.21-1.80)	0.371	0.119	0.501	0.52 (0.18-1.56)	0.247	0.223	0.730	0.58 (0.32-1.04)	0.069	0.082	0.260
3	UBE2E2	23413299	rs1496653	A	G	G	0.114	469	0.60 (0.33-1.09)	0.095	0.084	0.330		0.56 (0.29-1.05)	0.073	0.033	0.130	0.60 (0.12-2.92)	0.526	0.158	0.573	0.54 (0.07-4.50)	0.569	0.371	0.858	0.56 (0.28-1.11)	0.097	0.108	0.371
3	IGF2BP2	185793899	rs4402960	G	T	T	0.253	469	1.39 (0.96-2.01)	0.085	0.077	0.291		1.36 (0.85-2.18)	0.197	0.076	0.379	2.07 (0.95-4.51)	0.067	0.030	0.114	2.26 (0.96-5.29)	0.061	0.076	0.230	1.23 (0.74-2.04)	0.428	0.314	0.845
3	IGF2BP2	185795604	rs7651090	A	G	G	0.248	467	1.38 (0.94-2.02)	0.096	0.084	0.333		1.35 (0.84-2.18)	0.215	0.081	0.403	2.08 (0.87-4.97)	0.099	0.043	0.180	2.28 (0.93-5.58)	0.073	0.086	0.277	1.23 (0.75-2.04)	0.412	0.311	0.841
4	WFS1	6288259	rs4458523	T	G	T	0.315	463	0.80 (0.54-1.17)	0.250	0.176	0.764		0.92 (0.57-1.49)	0.730	0.198	0.632	0.31 (0.09-1.05)	0.060	0.027	0.101	0.32 (0.09-1.11)	0.073	0.086	0.278	1.06 (0.65-1.74)	0.820	0.438	0.858
5	ANKRD55	56510924	rs459193	A	G	A	0.234	467	1.35 (0.94-1.94)	0.108	0.094	0.378		1.29 (0.80-2.09)	0.304	0.108	0.485	2.18 (0.99-4.80)	0.054	0.025	0.089	2.26 (1.00-5.11)	0.050	0.064	0.185	1.11 (0.66-1.88)	0.695	0.405	0.858
5	PCSK1	96393194	rs6235	C	G	G	0.194	469	0.74 (0.48-1.16)	0.196	0.149	0.641		0.85 (0.51-1.42)	0.539	0.162	0.595	0.10 (0.01-1.79)	0.118	0.049	0.218	0.10 (0.01-1.80)	0.118	0.125	0.447	1.01 (0.61-1.69)	0.965	0.468	0.858
6	CDKAL1	20686765	rs9368222	C	A	A	0.226	469	1.50 (1.03-2.20)	0.036	0.035	0.107		1.81 (1.13-2.90)	0.014	0.011	0.022	1.17 (0.46-2.99)	0.746	0.199	0.627	1.50 (0.55-4.15)	0.430	0.314	0.846	1.86 (1.13-3.05)	0.015	0.032	0.063
6	RSPO3	127131790	rs2745353	C	T	C	0.401	469	0.80 (0.55-1.15)	0.223	0.164	0.705		0.65 (0.40-1.05)	0.081	0.036	0.149	1.00 (0.52-1.92)	0.996	0.245	0.666	0.76 (0.37-1.57)	0.465	0.331	0.852	0.62 (0.37-1.05)	0.073	0.086	0.279
7	DGKB	15024684	rs2191349	G	T	T	0.410	468	1.42 (0.99-2.03)	0.058	0.054	0.184		1.71 (0.99-2.96)	0.056	0.026	0.092	1.39 (0.74-2.62)	0.308	0.106	0.459	1.93 (0.91-4.07)	0.085	0.097	0.324	1.65 (0.93-2.92)	0.085	0.097	0.324
7	GRB10	50690548	rs933360	C	T	C	0.364	468	1.38 (0.99-1.93)	0.061	0.056	0.197		1.38 (0.84-2.27)	0.201	0.077	0.386	1.81 (0.99-3.31)	0.056	0.026	0.092	1.99 (1.02-3.90)	0.045	0.059	0.167	1.20 (0.70-2.05)	0.504	0.349	0.857
9	GLIS3	4287466	rs7041847	A	G	G	0.421	468	0.70 (0.49-1.00)	0.051	0.049	0.159		0.59 (0.36-0.96)	0.034	0.017	0.052	0.72 (0.36-1.44)	0.356	0.117	0.491	0.55 (0.26-1.15)	0.112	0.121	0.425	0.61 (0.36-1.02)	0.058	0.074	0.220
9	GLIS3	4293150	rs10814916	A	C	A	0.465	466	0.74 (0.52-1.04)	0.085	0.077	0.290		0.76 (0.46-1.26)	0.291	0.105	0.476	0.54 (0.27-1.05)	0.070	0.031	0.121	0.50 (0.24-1.04)	0.064	0.078	0.244	0.88 (0.52-1.50)	0.633	0.387	0.858
10	CUBN	17114152	rs1801222	A	G	A	0.262	468	0.83 (0.55-1.24)	0.366	0.233	1.000		0.68 (0.41-1.10)	0.116	0.050	0.231	1.57 (0.64-3.84)	0.326	0.112	0.472	1.28 (0.51-3.21)	0.594	0.374	0.858	0.60 (0.35-1.01)	0.054	0.069	0.201
10	ADRA2A	111282335	rs10885122	T	G	T	0.183	469	1.31 (0.87-1.96)	0.195	0.149	0.639		1.09 (0.67-1.79)	0.718	0.197	0.631	3.69 (1.52-8.95)	0.004	0.010	0.011	3.54 (1.38-9.07)	0.009	0.032	0.045	0.86 (0.50-1.50)	0.605	0.375	0.858
11	DUSP8	1675619	rs2334499	C	T	T	0.455	467	1.33 (0.96-1.85)	0.090	0.080	0.309		1.28 (0.75-2.17)	0.370	0.124	0.526	1.71 (1.00-2.91)	0.050	0.024	0.082	1.77 (0.93-3.36)	0.080	0.093	0.305	1.06 (0.60-1.90)	0.836	0.443	0.858
11	CRY2	45851540	rs11605924	A	C	C	0.499	469	0.72 (0.51-1.01)	0.057	0.054	0.182		0.84 (0.50-1.41)	0.505	0.158	0.584	0.45 (0.24-0.84)	0.012	0.010	0.022	0.46 (0.22-0.98)	0.044	0.059	0.164	1.05 (0.60-1.84)	0.863	0.443	0.858
11	MTNR1B	92940662	rs1387153	C	T	T	0.174	468	1.61 (1.06-2.43)	0.025	0.025	0.074		1.65 (1.00-2.71)	0.049	0.023	0.078	2.53 (0.84-7.68)	0.101	0.043	0.183	2.91 (0.94-8.97)	0.063	0.078	0.238	1.53 (0.91-2.58)	0.109	0.120	0.415
12	P2RX2	132465032	rs10747083	G	A	G	0.259	469	0.80 (0.53-1.21)	0.298	0.198	0.868		0.67 (0.41-1.10)	0.113	0.049	0.226	1.40 (0.54-3.62)	0.482	0.147	0.556	1.16 (0.44-3.04)	0.762	0.423	0.858	0.61 (0.36-1.03)	0.065	0.078	0.245
13	PDX1	27917061	rs2293941	G	A	A	0.295	469	1.09 (0.76-1.54)	0.647	0.364	1.000		1.50 (0.92-2.43)	0.103	0.045	0.201	0.46 (0.17-1.20)	0.110	0.047	0.203	0.61 (0.22-1.65)	0.325	0.264	0.802	1.79 (1.09-2.96)	0.022	0.033	0.086
19	GIPR	45693376	rs2302593	C	G	C	0.414	467	1.48 (1.06-2.07)	0.020	0.021	0.061		1.79 (1.04-3.07)	0.034	0.017	0.051	1.62 (0.92-2.85)	0.096	0.042	0.173	2.18 (1.11-4.28)	0.024	0.035	0.091	1.64 (0.92-2.91)	0.091	0.102	0.346
21	BACE2	41209710	rs737288	G	T	T	0.208	467	1.21 (0.82-1.79)	0.329	0.212	0.934		1.04 (0.63-1.72)	0.869	0.227	0.634	2.56 (1.07-6.08)	0.034	0.016	0.054	2.43 (1.01-5.86)	0.049	0.064	0.181	0.84 (0.48-1.46)	0.540	0.361	0.858
21	BACE2	41211811	rs6517656	G	A	A	0.178	468	1.24 (0.84-1.84)	0.277	0.190	0.824		1.15 (0.69-1.92)	0.584	0.171	0.608	2.13 (0.87-5.23)	0.100	0.043	0.182	2.12 (0.85-5.27)	0.107	0.118	0.408	0.98 (0.56-1.73)	0.951	0.466	0.858

Table that summarizes the most relevant results of the analysis of SNPs + Group models in Hispanic (HIS) ethnicity. ADD, Additive model; DOM, dominant model; REC, recessive model; HETHOM, heterozygous-homozygous model; CHROM, Chromosome code; LOCUS, Locus/Gene; POS, Base-pair coordinate [GRCh38]; ID, Variant ID; REF, Reference allele; ALT, Alternate allele; A1, Counted allele in logistic regression; A1_FREQ, minor allele frequency; OBS_CT, Number of samples in the regression; OR_CI95, odds ratio with 95% confidence interval.

#### General findings and effect of the nutritional intervention

Of a total of 110 variants included in the study, 98 (89.1%) passed the quality control. Of these, 40 (40.8%) presented some kind of significant association with GDM in at least one of the genetic tests considered, that is, the corresponding threshold was reached in some assessment criteria, with the following distribution by ethnicity: 13 (32.5%) only in the Caucasian ethnic stratum, 19 (47.5%) only in the Hispanic ethnic stratum and 8 (20.0%) in both ethnic strata (**Table 3a** CAU, **3b** HISP). The nutritional intervention presented a significant association with GDM, regardless of the variant considered; we obtained an OR < 1 for GROUP variable in favor of MedDiet, with all the significance criteria satisfied in practically all the tests of each model (**Supplementary Tables 1**CAU and **1**HIS).

#### Caucasian ethnicity findings

**Table 3a** CAU summarizes the most relevant findings for Caucasian pregnant women. The genetic variants significantly associated with increased risk of GDM were rs4402960, rs7651090, IGF2BP2; rs1387153, rs10830963, rs10830962, MTNR1B; rs17676067, GLP2R, rs1371614, DPYSL5; rs5215, KCNJ11; and rs2293941, PDX1. Variants significantly associated with decreased risk of GDM were rs780094, GCKR; rs7607980, COBLL1; rs3746750, SLC17A9; rs6048205, FOXA2; rs7041847, rs7034200, rs10814916, GLIS3; rs3783347, WARS; and rs1805087, MTR.

#### Hispanic ethnicity findings

**Table 3b** HIS summarizes the most relevant findings for Hispanic pregnant women. The genetic variants significantly associated with increased risk of GDM were rs9368222, CDKAL1; rs2302593, GIPR; rs10885122, ADRA2A; rs1387153, MTNR1B; rs737288, BACE2; rs1371614, DPYSL5; and rs2293941, PDX1. Variants significantly associated with decreased risk for GDM were rs340874, PROX1; rs2943634, IRS1; rs7041847, GLIS3; rs780094, GCKR; rs563694, G6PC2; and rs11605924, CRY2.

OR and *p* and *q-*values can be seen in the tables.

#### Additional findings

There are some variants for which some indication of association with GDM was obtained, but the results were not conclusive. Specifically, for CAU we can point to variants rs2785980 (LYPLAL1), rs7708285 (ZBED3) and rs573904 (SARDH), while for HIS we can point to variants rs1496653 (UBE2E2), rs4402960 (IGF2BP2), rs7651090 (IGF2BP2), rs4458523 (WFS1), rs459193 (ANKRD55), rs6235 (PCSK1), rs2745353 (RSPO3), rs2191349 (DGKB), rs933360 (GRB10), rs10814916 (GLIS3), rs1801222 (CUBN), rs2334499 (DUSP8), rs10747083 (P2RX2) and rs6517656 (BACE2) (**Table 3a** CAU and **Table 3b** HISP).

#### Bioinformatics analysis results

**Figure 2:**
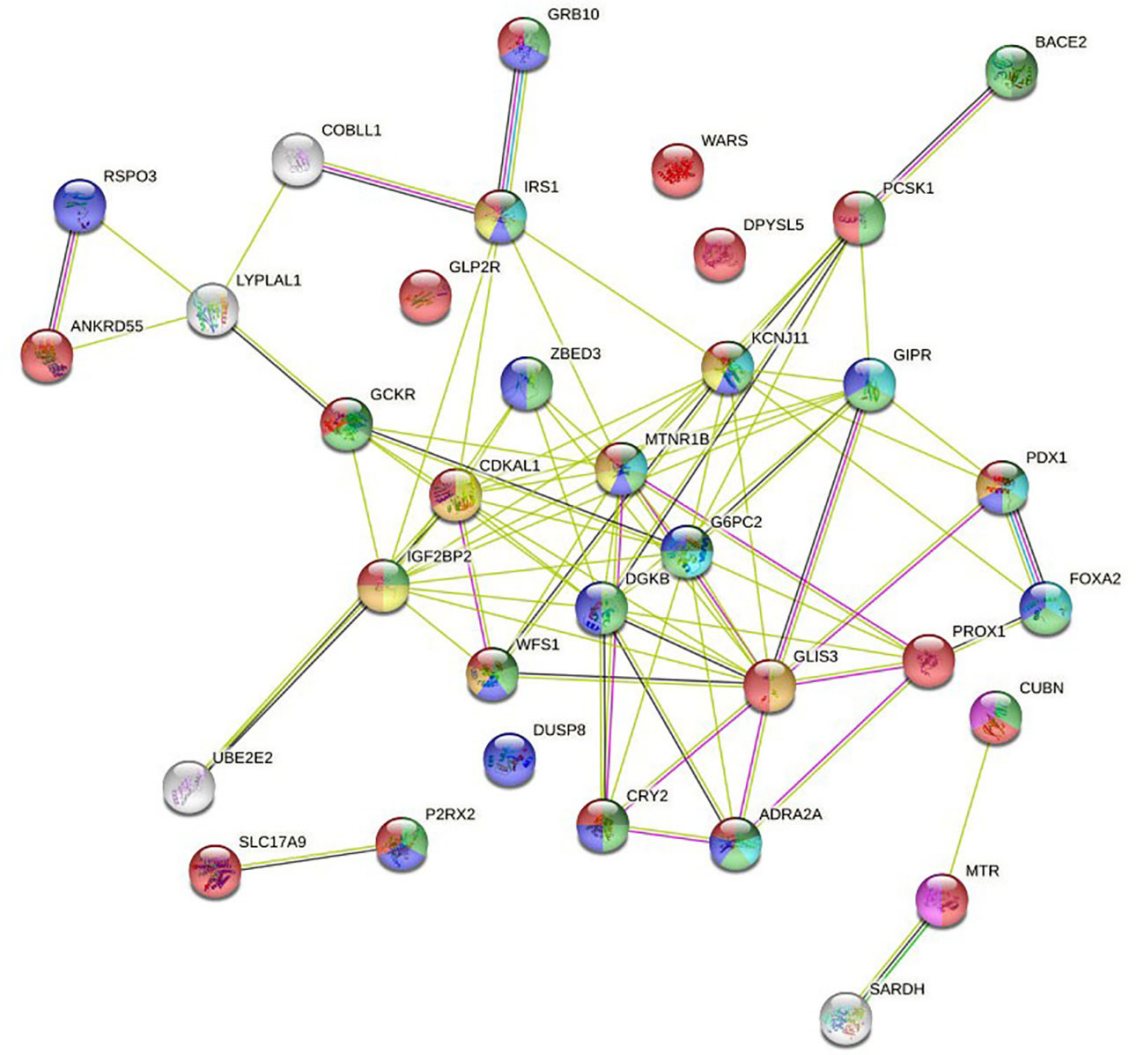
Full STRING network of both functional and physical protein associations. The edges indicate both functional and physical protein associations.

The 40 variants that presented some type of association with GDM were mapped to the closest gene/locus, resulting in a total of 34 encoding proteins that were used as STRING input data (**Supplementary Table 3**). Basic settings of analysis were: full STRING network, edges indicate both functional and physical protein associations, evidence as meaning of network edges, all active interaction sources, medium confidence (0.400) as minimum required interaction score. The complete results provided by the software can be found in **Supplementary Table 4**. The aspects that were considered most relevant to the objective of the work were selected by inspection so that **Supplementary Table 5**. **Table 4** displayed the bioinformatic analysis of relevant results, and the graph in **Figure 2** were obtained.

**Table 4:** Bioinformatic analysis relevant results.

QueryIndex	QueryItem	StringId	Disease	Diabetes Mellitus	Gestational Diabetes	Regulation of Biological Quality	Regulation of cell Communication	Glucose Homeostasis	Regulation of Insulin Secretion	Cobalamin
1	ADRA2A	9606.ENSP00000280155	**√**			**√**	**√**	**√**	**√**	
2	ANKRD55	9606.ENSP00000342295	**√**							
3	BACE2	9606.ENSP00000332979				**√**		**√**		
4	CDKAL1	9606.ENSP00000274695	**√**	**√**	**√**					
5	COBLL1	9606.ENSP00000341360								
6	CRY2	9606.ENSP00000478187	**√**			**√**	**√**	**√**		
7	CUBN	9606.ENSP00000367064	**√**			**√**				**√**
8	DGKB	9606.ENSP00000385780				**√**	**√**			
9	DPYSL5	9606.ENSP00000288699	**√**							
10	DUSP8	9606.ENSP00000380530					**√**			
11	FOXA2	9606.ENSP00000400341				**√**	**√**		**√**	
12	G6PC2	9606.ENSP00000364512				**√**	**√**	**√**	**√**	
13	GCKR	9606.ENSP00000264717	**√**			**√**		**√**		
14	GIPR	9606.ENSP00000467494				**√**	**√**		**√**	
15	GLIS3	9606.ENSP00000371398	**√**	**√**						
16	GLP2R	9606.ENSP00000262441	**√**			** **				
17	GRB10	9606.ENSP00000381793	**√**			**√**	**√**			
18	IGF2BP2	9606.ENSP00000371634	**√**	**√**	**√**	**√**				
19	IRS1	9606.ENSP00000304895	**√**	**√**	**√**	**√**	**√**	**√**	**√**	
20	KCNJ11	9606.ENSP00000345708	**√**	**√**	**√**	**√**	**√**	**√**	**√**	
21	LYPLAL1	9606.ENSP00000355895								
22	MTNR1B	9606.ENSP00000257068	**√**	**√**	**√**	**√**	**√**	**√**	**√**	
23	MTR	9606.ENSP00000355536	**√**							**√**
24	P2RX2	9606.ENSP00000343339	**√**			**√**	**√**			
25	PCSK1	9606.ENSP00000308024	**√**			**√**				
26	PDX1	9606.ENSP00000370421	**√**	**√**		**√**	**√**	**√**	**√**	
27	PROX1	9606.ENSP00000355925	**√**							
28	RSPO3	9606.ENSP00000349131					**√**			
29	SARDH	9606.ENSP00000360938								
30	SLC17A9	9606.ENSP00000359376	**√**							
31	UBE2E2	9606.ENSP00000379931								
32	WARS	9606.ENSP00000347495	**√**							
33	WFS1	9606.ENSP00000226760	**√**	**√**		**√**	**√**	**√**		
34	ZBED3	9606.ENSP00000255198	** **	** **	** **	**√**	**√**	** **	** **	** **

## Discussion

In this study, we have evaluated the association of 98 susceptibility genetic variants with the diagnosis of GDM in a large population of pregnant women from two ethnic groups, from a single center, living in Spain, in the setting of an ongoing nutritional intervention program. To our knowledge, this is the first time that a large relevant set of SNPs has been analyzed in such a large sample of GDM patients, and with a close follow-up regarding their diet and lifestyle.

We have observed that the nutritional intervention presented a significant association with GDM, regardless of the variant considered, OR < 1 (*p* < 0.05, *q <*0.05, *lfdr* < 0.1), in practically all models for both ethnicities [**Supplementary Table 2** CAU-2HIS], confirming the protective effect of the MedDiet for GDM, as previously reported (3, 4, 16, 17) and, at the same time, confirming the significance of the observed SNPs. The variable of the logistic regression model that represents the nutritional group [GROUP] provided relevant information to assess the association of the genetic variants with GDM. The analysis showed that the SNP-GDM association tests identified as significant, when adjusted by the GROUP variable, had a lower FDR, that is, the discoveries have a low proportion of false significant identified associations, evaluated by *q*-values, and a low local false discovery rate, evaluated by *lfdr*-values. Furthermore, *q*-values indicate that it is possible to qualify as discovery a null hypothesis with a *p*-value greater than the usual threshold of 0.05, increasing the set of variants that deserve further investigation, without significantly increasing the false discovery rate.

Although case-control-based GWAS usually refer to the additive model, it is currently recommended to also consider other genetic models (18) for a better understanding of the variant-disease relationship. Our study includes four genetic models that provide joint information on this relationship, aiding in the understanding of genetic analysis and providing further strengths to our findings. We can point out that, with some minor exceptions, when a significant association is observed for a given SNP in several models, the corresponding OR verify OR_ADD_ < OR_DOM_ < OR_REC_ < OR_HOM_, when minor allele is a risk allele or OR_ADD_ > OR_DOM_ > OR_REC_ > OR_HOM_ when minor allele is protective (**Table 3a** CAU-**3b** HIS).

Logistic regression results are consistent with information collected on STRING databases relative to PPI, both known and predicted, or associations identified by co-expression, protein homology, or text mining. The most significant variants in genetic tests are located in locus/genes encoding proteins annotated in the knowledge database as associated with biological processes related to diabetes and GDM (**Table 4**). Most of the nodes in **Figure 2** have the name of a locus/gene that are well referenced in the literature because several SNPs with a significant association with diabetes and GDM are located nearby. Specifically, the nodes located in the central core of the graph, MNTR1B (rs1387153, rs10830962, rs10830963), IGF2BP2 (rs4402960, rs7651090), KCNJ11 (rs5215), GCKR (rs780094), CDKAL1 (rs9368222), IRS1 (rs2943634), ADRA2A (rs10885122), CRY2(rs11605924), DKGB (rs2191349), G6PC2 (rs563694), GLIS3 (rs7041847, rs7034200, rs10814916), GIPR (rs2302593), WFS1 (rs4458523), ZBED3 (rs7708285), PROX1 (rs340874), FOXA2 (rs6048205), PDX1 (rs2293941), PCSK1 (rs6235), have been referred in various GWAS as associated to diabetes (6, 19–25), GDM (26–34) or both (35, 36).

We can observe a subnetwork made up of the RSPO3 (rs2745353), ANKRD55 (rs459193), LYPLAL1 (rs2785980) and COBLL1 (RS7607980) nodes. Although this is not annotated in STRING gene ontology, the revised literature reports that all of them are related to fasting insulin and show a significant association with diabetes and GDM (6, 19, 21–23, 35, 36).

In addition to the central core, where the nodes with the highest intensity of interaction are located, the network has three terminal nodes, four isolated nodes, and two isolated subnetworks, one made up of two nodes and the other made up of three nodes.

BACE2 (rs737288, rs6517656) node has been associated with GDM in some studies (8, 37), but not in others (28, 38). It is related to higher fasting C-peptide levels. As can be seen in the graph, it has a close interaction with PCSK1. In our work, the association for the Hispanic ethnic stratum is significant. GRB10 (rs933360) node has strong interaction with the IRS1 node, an insulin receptor substrate 1 that may mediate the control of various cellular processes by insulin. It is associated with diabetes in some studies (32–34), and with both diabetes and GDM in other (35, 36). We have found an association with GDM in the Hispanic ethnic stratum. UBE2E2 (rs1496653) node is an ubiquitin-conjugating enzyme associated with diabetes in some reports (20, 21, 25), and with GDM in other studies (27, 35, 36). In our work, it shows interaction with IGF2BP2, but it barely reaches significance in the Hispanic ethnicity.

DPYSL5 (rs1371614) has been associated with diabetes (6, 23, 24) and GDM (35, 36). It is a dihydropyrimidinase-related protein that has been linked with fasting glucose. In our study, we found an association in some models for both ethnic groups. WARS (rs3783347) is a shear stress-responsive gene that has been associated with diabetes (19, 22–24). In our study, it is significant in some models for Caucasian ethnicity. DUSP8 (rs2334499), dual specificity protein phosphatase 8, has phosphatase activity with synthetic phosphatase substrates and negatively regulates mitogen-activated protein kinase activity. Some studies (20, 21) report association with diabetes, while others (27, 36) do so with GDM. Our work shows association in a model for Hispanics. GLP2R (rs17676067) is a receptor for glucagon-like peptide 2, which has been reported as associated with diabetes (21). Our work shows association in the ADD, REC and HOM models for Caucasian ethnicity.

SLC17A9 (rs3746750), Solute Carrier Family 17 Member 9, is a protein coding gene related with transporter activity and involved in vesicular storage and exocytosis of ATP. It has been related to purinergic signaling and diabetes (39, 40). In our work, it shows a significant association in the ADD, DOM and HET models for Caucasian ethnicity. In the graph, we can see a strong association of SLC17A9 with P2RX2 (rs10747083), purinoceptor 2, ion channel gated by extracellular ATP involved in a variety of cellular responses. It is included in some studies as associated with diabetes (19, 22, 23) and GDM (36). In our study, it hardly reaches significance in the DOM model of the Hispanic ethnicity.

The CUBN (rs18001222), MTR (rs1805087), and SARDH (rs573904) proteins define a subnetwork in the graph that play a role in one-carbon metabolism with functions in many cellular processes. Also, genetic variants in the transport and metabolism of folate modify glycemic control and risk of GDM, and the effect of folic acid on homocysteine levels is modulated by CUBN (rs1801222) (41). CUBN, cubilin, is a cotransporter which plays a role in lipoprotein, vitamin and iron metabolism; serves as transporter in several absorptive epithelia, including embryonic yolk sac. In a study by Böger et al. (42) it is described as “*a gene locus for albuminuria”*, an idea that is reiterated in subsequent works (43). It has also been associated with type 2 diabetes in an elderly population (44). In our work, it is in the limits of significance in the DOM and HET model in the Hispanic ethnicity. MTR, 5 -methyltetrahydrofolate–homocysteine ​​methyltransferase, catalyzes the transfer of a methyl group from methyl- cobalamin to homocysteine; belongs to the vitamin-B12 dependent methionine synthase family, and has been associated with various biological processes related to pregnancy (45). In our work, it has been significant in the ADD model for Caucasian ethnicity.

It should be noted that some studies are partially in disagreement with the most widely accepted results, that is, they report no association with diabetes or GDM in some of the variants mentioned above. In this regard, the following works can be consulted (9, 38, 46–49):. As an example, in our study some SNPs included in the initial list of variants and clearly identified in the literature, such as TCFL2, KCNQ1, HNFA1A, SCL30A8, have not reached a level of significance in any association model with GDM. This could be related to the complex genetic and epigenetic architecture, with both similarities and differences between diabetes and GDM, which deserves further investigation.

The idea of considering the evaluation of the impact of diet and lifestyle on the significance of SNPs in their association with GDM is currently attracting the interest of investigators (50). In this regard, we remark that our study has been performed with a meticulous evaluation of lifestyle habits, showing the protective effect of a healthy MedDiet, and that significant SNPs remained as such, after performing a rigorous genetic and statistical bioinformatic analysis.

## Conclusion

Identifying the potential susceptibility genetic variants that could be associated with developing GDM and their modulation due to a nutritional intervention deems useful to design preventive and therapeutic strategies, especially in the setting of the increasing prevalence of GDM. In this study, we have examined a set of 98 SNPs in a large cohort of patients from two main ethnicities from a single center, and in the setting of an ongoing clearly beneficial nutritional intervention. The study confirms previous works that promote the therapeutic recommendation of Mediterranean Diet to all pregnant women to prevent GDM. In addition, we have confirmed a core set of SNPs reported in the literature as associated with diabetes and GDM. However, our statistical models, that include the nutritional intervention as an additional variable, highlight and reinforce the significance of the association effects, reducing the FDR levels. This means that a safer tool is available to control the risk of GDM based on the genomic profile of the individual. Therefore, genotypic analysis of women of child-bearing age and recommending a MedDiet, will assist the prompt identification and management of GDM.

## Data availability statement

The original contributions presented in the study are included in the article/**Supplementary Material**. Further inquiries can be directed to the corresponding authors.

## Ethics statement

The studies involving human participants were reviewed and approved by the Clinical Trials Committee of the Hospital Clínico San Carlos. The patients/participants provided their written informed consent to participate in this study.

## Author contributions

Conceptualization and design: AR-L, AB, AC-P, NT, ADu, MH, MR, LM, MZ, PM, ADi, LV, VM, JV. Data curation, and analysis and interpretation of data: AR-L, AB, AC-P, NT, ADu, CF, IJ, LV, VM, IM, JV. Funding acquisition: AC-P, NT. Investigation: AC-P, MT, PM, ADi, AB, MA, LS, LM, MZ, MR, MT. Methodology: AC-P, NT, ADu, CF, IJ, MH, MT, IM, PM, MA, LS, LM, MZ, AB, LV, VM, JV. MR. Software: AR-L. Supervision, Validation and Visualization: AC-P, AR-L, AB, NT, MR. Writing – original draft: AC-P, AR-L, AB, NT. Writing – review & editing: AR-L, AC-P, AB, NT, ADu, MR, MA, LS, LM, MZ. All authors have seen and agree with the content of the full last version of manuscript.

## Funding

This research was funded by grants from the Instituto de Salud Carlos III/MICINN of Spain under grant number PI20/01758, and European Regional Development Fund (FEDER)’’A way to build Europe’’ and Ministerio de Ciencia e Innovación, and Agencia Estatal de Investigación of Spain under grant number PREDIGES RTC2019-007406-1.The design and conduct of the study; collection, management, analysis, and interpretation of the data; preparation, review, and approval of the manuscript; and decision to submit the manuscript for publication are the responsibilities of the authors alone and independent of the funders.

## Acknowledgments

We wish to acknowledge our deep appreciation to the administrative personnel and nurses and dieticians from the Laboratory Department (Marisol Sanchez Orta, María Dolores Hermoso Martín, María Victoria Saez de Parayuelo), the Pregnancy and Diabetes Unit and to all members of the Endocrinology and Nutrition and Obstetrics and Gynecology departments of the San Carlos Clinical Hospital and the Central Unit for Research in Medicine (UCIM),University of Valencia, Valencia, Spain.

## Conflict of interest

LM, MZ, LS, MA are employees of Patia Europe.

The remaining authors declare that the research was conducted in the absence of any commercial or financial relationships that could be construed as a potential conflict of interest.

## Publisher’s note

All claims expressed in this article are solely those of the authors and do not necessarily represent those of their affiliated organizations, or those of the publisher, the editors and the reviewers. Any product that may be evaluated in this article, or claim that may be made by its manufacturer, is not guaranteed or endorsed by the publisher.
